# Evaluation of the anticancer effects of hydroxycinnamic acid isomers on breast cancer stem cells

**DOI:** 10.1007/s12032-025-02618-3

**Published:** 2025-02-11

**Authors:** Tülin Burhanoğlu, Zehra Seda Halbutoğulları, Gulseren Turhal, Asuman Demiroglu-Zergeroglu

**Affiliations:** 1 Department of Chemistry, Faculty of Science, Gebze Technical University, Kocaeli, Turkey; 2 Department of Molecular Biology and Genetics, Faculty of Science, Gebze Technical University, Kocaeli, Turkey; 3 Center for Stem Cell and Gene Therapies Research and Practice, Kocaeli University, Kocaeli, Turkey; 4 Department of Medical Biology, Faculty of Medicine, Kocaeli University, Kocaeli, Turkey

**Keywords:** Breast cancer stem cell, Ortho-coumaric acid, Para-coumaric acid, Anti-cancer effect, Apoptosis, Metastasis

## Abstract

Research on breast cancer stem cells (BCSCs) is crucial for improving our understanding of their roles in tumor resistance, metastasis, and relapse. This study investigated the anti-cancer effects of two isomers of hydroxycinnamic acids (HCA): para-coumaric acid (PCA) and ortho-coumaric acid (OCA) on breast cancer stem cells (BCSCs). The isolated and characterized stem cells contained CD44 + /CD24 surface markers, exhibited high levels of aldehyde dehydrogenase activity, and were able to form mammospheres. The evaluation of HCAs on stem cell proliferation, cell cycle, and apoptosis was conducted by comparing them with MCF-7, the luminal breast cancer cell line. The viability and immunoblot analyses demonstrated that HCA applications resulted in a dose-dependent decrease in the number of viable cells and inhibited phosphorylation of Extracellular regulated kinases 1/2 (ERK1/2). These findings were supported by the detection of suppressed colony formation and delayed wound-healing in HCA-exposed cells. E-cadherin expression increased in OCA-treated cells. Additionally, the arrest of G1/S phase progression and the downregulation of Cyclin D1 expression exhibited that OCA and PCA-induced cytostatic effects in BSCS cells. After treatment, the increased Annexin-V/7-AAD staining, along with elevated expression of caspase-3/7 and a decreased Bcl-2/Bax ratio, indicated apoptosis mediated by the activation of Janus kinase (JNK) and p38 Mitogen-activated kinase (p38 MAPK). In conclusion, both OCA and PCA exhibit anti-carcinogenic potential on BCSCs; However, OCA has a stronger effect and is becoming a promising candidate for further research.

## Introduction

Breast cancer is the most common cancer among women globally, with over 2.3 million new cases diagnosed in 2022, leading to 670,000 fatalities [1]. Breast cancer treatment encompasses local methods such as surgery and radiotherapy, as well as systemic approaches such as chemotherapy, hormone therapy, and targeted therapy [2]. Despite these treatments, some patients experience recurrence or metastasis due to breast cancer stem cells (BCSCs), a specific subset of tumor cells. BCSCs are the focus of cancer research because of their self-renewal capacity, high proliferation rate, and ability to generate diverse cancer cell lineages that contribute to treatment resistance [3]. BCSCs were identified and isolated using the CD44 + /CD24 − (dim)/Lin − surface marker phenotype [4]. CD44 is crucial for adhesion, migration, and invasion in breast cancer because it promotes cell proliferation, tumor progression, and angiogenesis [5, 6]. CD24, a surface glycoprotein expressed at low levels, also positively affects tumor growth and metastasis [7]. Aldehyde dehydrogenase (ALDH) activity, another BCSC marker, is associated with self-renewal, colony formation, tumor initiation, and drug resistance, which are hallmarks of stemness in various cancers [8, 9].

Although chemotherapy remains the primary cancer treatment, there is growing emphasis on the development of new natural herbal antitumor drugs. This shift is driven by the emergence of resistance to chemotherapeutic agents and the significant side effects associated with these drugs [10, 11]. HCAs, comprising one-third of the phenolic acids found in plants, have attracted considerable interest owing to their anticancer properties and antioxidant potential [12, 13]. PCA, the most prevalent HCA isomer in nature, reduces cancer cell proliferation, induces cell cycle arrest, inhibits cell migration, promotes apoptosis, and exhibits antioxidant, immunomodulatory, and anti-inflammatory effects [14–17]. Furthermore, it has been demonstrated that this compound diminishes the cytotoxic effects of chemotherapeutic agents such as cisplatin and doxorubicin in healthy cells, protects against oxidative stress, inflammation, and apoptosis, and exerts anticancer effects by interacting with enzymes involved in the metabolism of carcinogens [14, 18]. These findings suggest that this compound could be a valuable adjuvant therapy in combination with chemotherapy [19]. Although some data indicate that OCA, another HCA isomer, has antioxidant properties, inhibits adipocyte differentiation, exhibits toxic effects on epithelial and salivary gland carcinoma cells, and induces apoptosis in MCF-7 cells, its anticancer potential remains limited [20–23].

This study aimed to explore the anticancer effects of two natural HCA isomers on breast cancer stem cells, given their significance in treatment resistance and prevention of disease recurrence. Our goal was to highlight their potential application in direct or adjuvant therapy. In this context, we examined the antiproliferative, cytostatic and apoptotic potentials of OCA and PCA using primary cultured BCSCs obtained from tumor tissues removed after mastectomy. Additionally, the anti-cancer effects of these compounds were compared with those of MCF-7, epithelial human breast cancer cells.

## Methods

### Compounds and cell culture

Para-coumaric acid (4-Hydroxycinnamic acid, HOC_6_H_4_CH) (C9008) and ortho-coumaric acid (2-Hydroxycinnamic acid, HOC_6_H_4_CH) (H22809) were purchased from Sigma-Aldrich. Both compounds were prepared as 4 M stock solutions in DMSO and stored at – 20 °C.

BCSCs were previously isolated from the primary breast tumor of a 57-year-old woman with stage two invasive ductal carcinoma after mastectomy according to the principles of the Declaration of Helsinki [24]. This study was approved by the Ethics Committee of Kocaeli University (Approval No: KU GOKAEK2018/357). The tumor cells were reported to have an estrogen receptor (ER) 100%, progesterone receptor (PR) 95%, and 11% Ki67 proliferation index. BCSCs were cultured in DMEM/F12 (Thermo Fisher Scientific 11,320,074) supplemented with 10% fetal bovine serum (FBS) (Gibco 10,270,106), 1% penicillin/streptomycin (Gibco 15,140,122), 1% glutamate (Gibco 35,050,061), 1 μg/ml hydrocortisone (Sigma H4001), 4 μg/ml insulin (Gibco 12,585,014), and 10 ng/ml Epidermal Growth Factor (EGF) (Prospec CYT217). MCF-7 cells were obtained from ATCC (Cat no: HTB-22) and cultured in DMEM/F12 (Thermo Fisher Scientific 11,320,074) supplemented with 10% FBS, 1% penicillin/streptomycin, 1% glutamate, and 10 μg/ml insulin. Both cell lines were cultured in a humidified incubator at 37 °C with 5% CO_2_.

### Characterisation of BCSCs

#### Determination of cell surface molecules

Flow cytometry was used to evaluate cancer stem cell surface antigens of cells isolated from the tumor tissue. At the end of the 3rd passage, the cells were harvested using 0.25% trypsin–EDTA, washed twice with PBS, and resuspended in PBS. A 100 µL cell suspension (1 × 10^6^ cells) was incubated with fluorescently labelled monoclonal antibodies for 30 min at room temperature in the dark. After washing with PBS, the cells were analysed using a FACSCalibur flow cytometer (BD Biosciences, San Jose, CA, USA). The same procedure was repeated at the 7th, 10th, 15th, and 20th passages to confirm the persistence of cancer stem cell characteristics. Monoclonal antibodies anti-CD44 (BD555478), anti-CD13 (347,406), anti-CD140b (558,821), anti-CD166 (559,263), anti-CD73 (550,257), anti-CD105 (560,839), anti-CD29 (555,443), anti-CD90 (555,596), anti-CD45 (555,482), anti-HLA-DR (555,558), anti-CD24 (555,428), anti-CD14 (345,785), anti-CD34 (333,178), anti-CD61 (564,173), anti-cytokeratin (550,953) and isotype controls; IgG1 FITC/IgG2a PE (342,409), IgG1k PerCP5.5 (550,795) were purchased from BD Biosciences, anti-CD133/1 (130,080,801) from Miltenyi Biotec, Germany.

#### Measurement of aldehyde dehydrogenase (ALDH) enzyme activity

ALDH activity was measured using an Aldeflour Assay Kit (StemCell Technologies, Vancouver, Canada). Cells were harvested and suspended in an assay buffer. To each 500 µL cell suspension (0.5 × 10^6^ cells), 2.5 µl of activated ALDEFLUOR™ reagent was added. 5 µL of ALDH inhibitor N, N-diethylaminobenzaldehyde (DEAB) was added as a negative control. The samples were incubated at 37 °C for 45 min and centrifuged at 300 × g for 5 min. The pellet was resuspended in 300 µL assay buffer and analysed using a FACSCalibur flow cytometer.

#### Mammosphere formation

The mammosphere formation ability of tumor cells were assessed to identify cancer stem cells. The 6-well plates were coated with 3% agar to prevent cell adhesion, and 1 × 10^5^ cells were seeded in 1 ml of mammosphere medium per well. The medium consisted of DMEM/F12, 1% penicillin/streptomycin, 1% GlutaMAX, 1 μg/ml hydrocortisone, 4 μg/ml insulin, 20 ng/ml EGF, and 20 ng/ml bFGF. The cells were cultured at 37 °C with 5% CO_2_ for seven days, after which the mammospheres were observed and photographed. After collection and centrifugation (300 × g for 5 min), the pellet was treated with 0.25% trypsin to obtain a single-cell suspension, which was reseeded at the same density for the second generation. The mammospheres formed in the second generation were also observed and photographed.

### Viability assay

To assess the effect of the compounds on cell viability, BCSCs and MCF-7 cells were plated in a 96-well plate at a density of 1 × 10^4^ cells/well and incubated at 37 °C for 24 h. The medium was replaced with a freshly prepared medium containing 0–40 mM OCA and PCA. After 72 h, the medium was discarded and 10% WST-1 (Roche Diagnostics, Germany) solution was added to the wells. After 2 h of incubation, absorbance was recorded at 480 nm using a microplate reader spectrophotometer. At least four wells were used for each dilution. The mean absorbance values were analysed, and IC_50_ values for each compound were calculated using GraphPad Prism v8.3 (GraphPad Software, Inc., San Diego, CA, USA) software.

### Wound-healing assay

To investigate the effect of the compounds on the growth, survival, and migration capacity of the cells, BCSCs and MCF-7 cells were cultured in 6-well plates until 80–90% confluency was reached. Confluent cells were scratched with a pipette tip to create a wound and then washed with PBS. Then, a medium containing 0, 2.5- 5 mM OCA, PCA, and 30 µM cisplatin was added to the wells. After incubation for 24 h, the wound area was examined and imaged under an inverted microscope. Images were analysed using the ImageJ software (https://imagej.nih.gov/ij/download.html). The wound areas that formed at the beginning and remained unclosed at the end of the experiment were calculated. The effects of the compounds were statistically analysed using the following wound closure formula:

$$Wound \, closure \, \% \, = \, \left( {wound \, area \, at \, the \, beginning \, {-} \, wound \, area \, remaining \, at \, the \, end \, of \, the \, time} \right)/wound \, area \, at \, the \, beginning \, \times \, 100$$

### Colony formation assay

To assess the effects of OCA and PCA on colony formation, BCSCs and MCF-7 cells were seeded in 6-well plates at a density of 1.6 × 10^3^ cells per well. After 24 h, the cells were observed to adhere to the culture surface, and the medium was replaced with media containing 2.5- 5 mM OCA or PCA, with 30 µM cisplatin as a control. After seven days of incubation, the colonies were fixed with 4% formaldehyde for 20 min and stained with 1.5% crystal violet for 5 min. To quantify colony formation, the colonies were extracted in 10% acetic acid, and the absorbance of the dye was measured at 595 nm. The effects of the compounds on colony formation were then analysed [25, 26].

### Cell cycle analysis

BCSCs (1.5 × 10^5^ cells) and MCF-7 cells (3 × 10^5^ cells) were seeded in 6-well plates and incubated for 24 h at 37 °C in 5% CO_2_. To synchronize the cell cycle, the medium was replaced with a serum-free medium and incubated for 24 h. Next, cells were treated with 5–10 mM OCA, PCA, or 30 µM cisplatin (positive control) for 24 h. After incubation, dead cells were collected from the medium and adherent cells were detached with 0.25% trypsin. Both were combined and washed twice with cold phosphate-buffered saline (PBS). The cells were fixed with 70% ethanol at 4 °C for 1 h and then stored at − 20 °C. After centrifugation and washing, 0.5 ml of the reagent (containing detergent, propidium iodide, and RNase A) provided in the cell cycle kit (Beckman Coulter CO3551) was added, and cells were incubated for 20 min in the dark. Cell cycle analysis was performed using a Navios flow cytometer (Beckman Coulter, Miami, FL, USA), and Kaluza 2.1 software was used to determine the distribution of cells in the sub-G_1_, G_1_/S, S, and G_2_/M phases.

### Apoptotic analysis

The induction of apoptosis by OCA and PCA was assessed by flow cytometry using an Annexin-V-7AAD apoptosis assay kit (Beckman Coulter, France). BCSCs (1.5 × 10^5^ cells) and MCF-7 cells (3 × 10 ^5^ cells) were seeded in 6-well plates and incubated for 24 h. After replacing the medium with 5–10 mM OCA or PCA, cells were incubated for another 24 h. At the end of the incubation period, both suspended dead and adherent cells (harvested with 0.25% trypsin–EDTA) were collected. Cells were washed with cold PBS, centrifuged, and resuspended in 100 µL binding buffer. After adding 10 µL annexin-V FITC and 20 µL 7AAD, the cells were incubated in the dark for 20 min. The reaction was stopped with 350 µL of assay buffer and the cells were analysed using a Navios flow cytometer.

### RT-PCR analysis

BCSCs (1.5 × 10^5^ cells/well) and MCF-7 cells (3 × 10^5^ cells/well) were seeded into 6-well plates for 24 h. Then, the medium was replaced with fresh medium containing 5 or 10 mM OCA or PCA, respectively. After an additional 24 h of incubation, the cells were harvested, and total RNA was isolated using the PureLink® RNA Mini Kit (Invitrogen, CA, USA) according to the manufacturer's instructions. cDNA was obtained using a cDNA Synthesis Kit (Applied Biosystems, CA, USA) and amplified using the RealQ Plus Master Mix Green Amplicon kit. RT-PCR was performed on a LightCycler II 480 (Roche, Mannheim, Germany). The primer sequences used are listed in Table 1. Changes in BAX, BCL-2, CASP3, and CASP7 levels were analysed to determine apoptosis, and the cell cycle and cell migration were assessed by the expression levels of CCND1 and CDH1 genes, respectively. Relative mRNA levels were normalized to those of the internal control HPRT1.

**Table 1:** Primer sequences of genes used in RT-PCR analysis

Gene name	Primer base sequence (5′ → 3′)
Caspase-3 (CASP3)	L- ttgtggaattgatgcgtgat R- ggctcagaagcacacaaaca
Caspase-7 (CASP7)	L- gctgacttcctcttcgccta R- caaaccaggagcctcttcct
B-cell lymphoma 2 (BCL-2)	L- gcacctgcacacctggat R- agggccaaactgagcaga
BCL2 associated X (BAX)	L- catcatgggctggacattg R- gggacatcagtcgcttcagt
Cyclin D1 (CCND1)	L- gtgaagttcaattccaatccgc R- gggacatcaccctcacttac
E-Cadherin I (CDH1)	L- aggggtctgtcatggaaggt R- gcggcattgtaggtgttca
Hypoxanthine phosphoribosyltransferase 1 (HPRT1)	L- tgaccttgatttattttgcatacc R- cgagcaagacgttcagtcct

### Western blot analysis

Cells were seeded in 6-well plates and treated with 5–10 mM OCA or PCA for 24 h. After scraping, cells were lysed with a solution containing protease phosphatase inhibitors. Protein concentration was measured using the Pierce BCA Protein Assay Kit (Thermo Fisher Scientific, Waltham, MA, USA). For SDS-PAGE, 30 µg of protein was loaded onto a 10% gel, and the proteins were transferred to a PVDF membrane. The membrane was blocked with 5% skimmed milk powder and incubated overnight with primary antibodies against phospho-ERK1/2 and total ERK1/2 (CST 8544; 9107), phospho-JNK and total JNK (CST 4668; 9252), and phospho-p38 and total p38 (CST 4511; 9212). The membrane was then washed and incubated with an anti-rabbit HRP-conjugated secondary antibody (CST 7075). Protein bands were detected using an ECL reagent (Advansta K-12045-D20) and visualized using ChemiDoc XRS + (Bio-Rad).

### Statistical analysis

The experiments were conducted thrice, and statistical analyses were performed using GraphPad Prism version 8.3. Dunnett’s multiple comparison test was used for One-way ANOVA, whereas Tukey’s multiple comparison test was applied for Two-way ANOVA. Differences between the control and experimental groups were considered significant at *p < 0.05 and highly significant at **p < 0.01.

## Results

### The cells isolated from tumor tissue carry the main characteristics of BCSCs

**Fig. 1:**
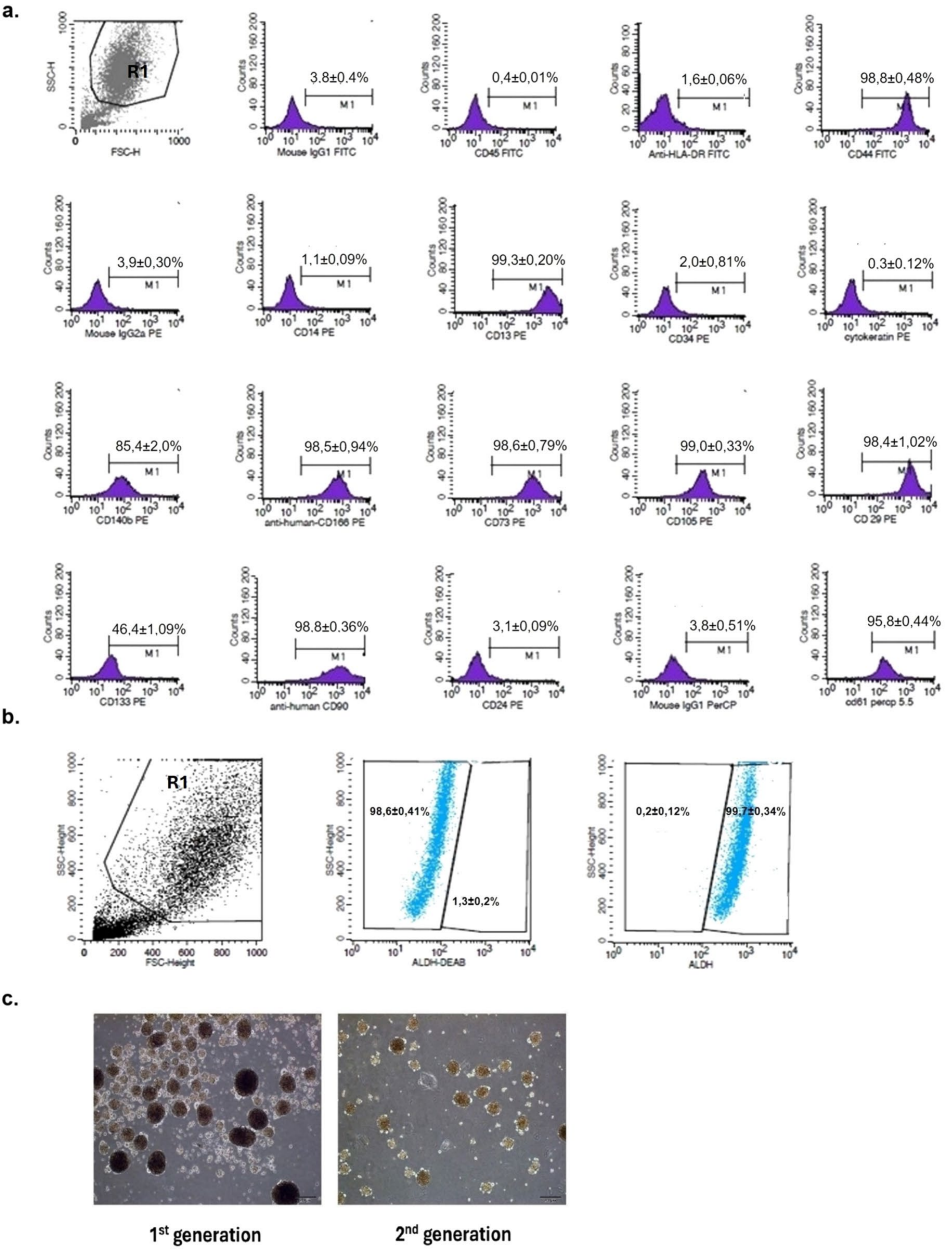
Characterisation of cells isolated from breast tumor (**a**)**.** Identification of cell surface antigens; cells were incubated with fluorescent conjugated monoclonal antibodies for 30 min and analysed with flow cytometry (n = 3) (**b**)**.** Assessment of ALDH enzyme activity through flow cytometry (n = 3) (**c**). Light microscopy images of 1st and 2nd generation mammospheres formed by BCSCs in suspension culture, with a scale bar of 200 µm. (n = 3) All experiments were conducted in triplicate, and the standard deviation was calculated

To identify breast cancer stem cells, breast tumor tissues were assessed for specific surface antigens, ALDH enzyme activity, and mammosphere formation. The debris and cell population were first visualized in a forward scatter side scatter (FS-SS) plot based on granularity and size during flow cytometry analysis. The cell population was gated and designated as R1, but low-size and low-granularity debris and dead cells were excluded. The fluorescence intensity of cells within gate R1 was measured using CellQuest software. Cells incubated with FITC, PE, and PerCP isotype controls were used as negative controls for immunofluorescence. The ratio of fluorescence-positive cells to the total cell population was then determined. Flow cytometry analysis revealed that the isolated cells showed a CD44^high^ + /CD24^−^/CD61^high+^ phenotype with 46.49% CD133 positivity. Additionally, as shown in Fig. 1a these cells showed a stem cell phenotype in terms of other CD molecules identified in stem cell characterization, positive for CD90, CD105, CD73, CD29, CD166, CD140b, and CD13, and negative for CD45, CD34, CD14, and HLA-DR [27]. An Aldefluor assay was conducted to assess ALDH enzyme activity, with DEAB used as a negative control to inhibit the enzyme. Flow cytometry results showed that 99.3% of the cells exhibited high ALDH activity compared to that of the negative control (Fig. 1b). Additionally, formation of mammosphere was observed in cultured suspensions (Fig. 1c). Furthermore, when these mammospheres were cultured again into single-cell suspension, they retained their mammosphere-forming properties during the second generation.

### The viability of BCSC cells was reduced after OCA and PCA treatments

**Fig. 2:**
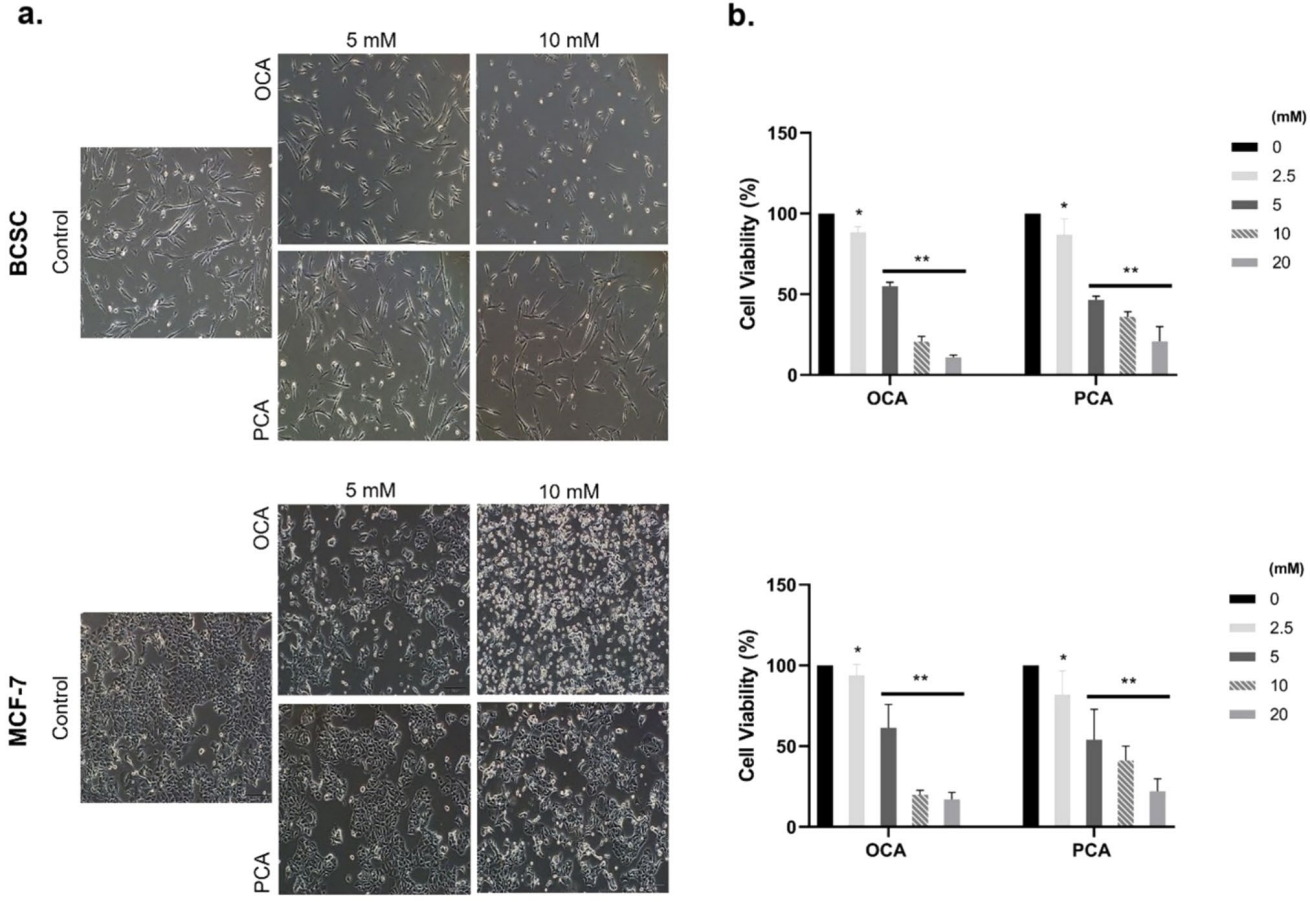
The effect of OCA and PCA on morphology and cell viability (**a**)**.** Light microscopy images of the cells are shown as follows: BCSCs and MCF-7 Cells were incubated with a medium containing OCA or PCA for 24 h (10 × magnification) (**b**)**.** A decrease in the viability of cells treated with the agents for 72 h was detected by the WST-1 proliferation assay (n = 3). All data are expressed as the mean ± SD of three independent experiments. Absorbance values were analysed with GraphPad Prism version 8.3 *p < 0.05, and **p < 0.01

To investigate the effects of OCA and PCA on cell viability, consecutive concentrations of OCA and PCA (0–40 mM) were applied to BCSCs and MCF-7 cells, and confluency and the viability of cells were analysed by microscopy and WST-1 assay, respectively. As shown in Fig. 2a, both agents decreased the number of cells compared with the control cells. In addition, altered morphology was observed in the cells exposed to 10 mM of OCA and PCA for 24 h. The IC_50_ values of OCA and PCA were calculated as 5.13 mM and 4.27 mM in BCSCs and 5.25 mM and 4.6 mM, respectively, in MCF-7 cells in 72 h (Fig. 2b).

### Delayed wound closure was observed in both types of cells exposed to HCAs

**Fig. 3:**
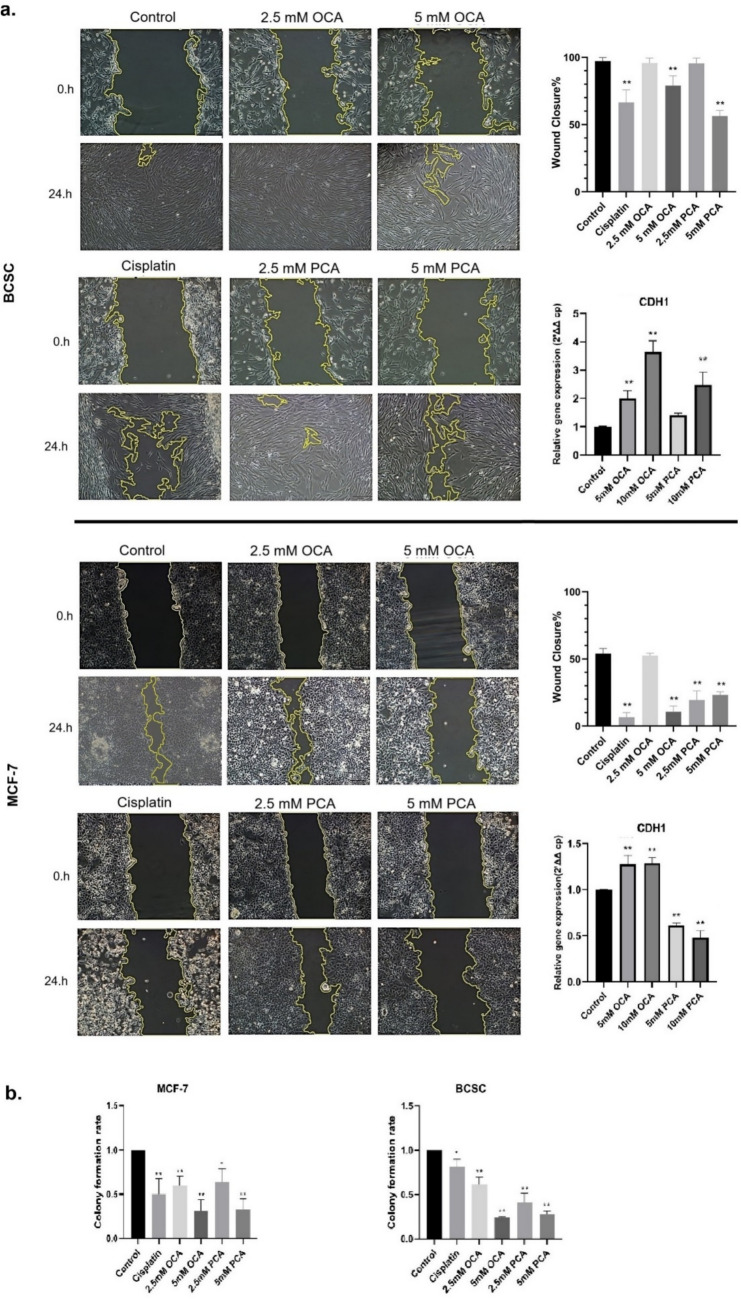
Assessment of wound-healing/migration and colony formation abilities of cells treated with HCAs (**a**)**.** Wound closure assay images (10 × magnification). The remaining wound areas at the end of 24 h were calculated using the ImageJ software. CDH1 gene expression levels of the cells treated with 5–10 mM concentrations of the compounds for 24 h (**b**)**.** Colonies of cells treated with compounds were stained with crystal violet at the end of 7 days, and the absorbance values were measured after extraction with acetic acid (n = 3). All data are expressed as the mean ± SD of three independent experiments and were analysed with GraphPad Prism version 8.3 *p < 0.05, **p < 0.01

To assess the impact of OCA and PCA on the healing of cells, a wound-healing assay was performed in 24 h. Cells were treated with 2.5 and 5 mM of either HCA, and the wound area was examined at the beginning and end of incubations. As demonstrated in Fig. 3a, compared to control cells, neither of the HCAs had a significant effect on the growth/migration of BCSCs at 2.5 mM concentration. However, treatments with either 5 mM OCA or PCA prevented the cell growth and migration of these cells. However, OCA or PCA was more effective in delaying healing in MCF-7 cells. Additionally, the CDH1 expression was examined in both cell lines after OCA and PCA treatment. CDH1 mRNA levels were elevated after OCA treatment starting at 5 mM in both cell lines. Interestingly, treatment with PCA either did not change or reduce the CDH1 levels in BCSC and MCF-7 cells, respectively (Fig. 3a). Overall, both compounds appeared to inhibit growth/migration in a concentration-dependent manner, and OCA and PCA caused different modulations in CDH1 expression, suggesting that they may have distinct mechanisms to inhibit growth/migration.

### Colony formation of cells was prevented after OCA and PCA treatments

To evaluate the effects of OCA and PCA on colony formation, single-cell colony formation assays were performed using BCSCs and MCF-7 cells. Both cell lines were seeded in 6-well plates at low density to ensure attachment to distinct locations and then treated with 2.5 and 5 mM concentrations of OCA or PCA. Following seven days of incubation, small and compact colonies were visible after staining with crystal violet in the control group; however, only a few colonies were observed in OCA- or PCA-treated cells for each HCA. 2.5 mM of OCA or PCA significantly reduced the number of both cells. For quantitative analysis of colony formation, cells were extracted with 10% acetic acid, absorbance was measured, and statistical analyses were performed using One-way ANOVA (Fig. 3b). This analysis revealed that 2.5 mM OCA and PCA reduced the colony number to approximately half of that observed in the control.

### OCA and PCA-induced G_1_/S arrest in both cell lines

**Fig. 4:**
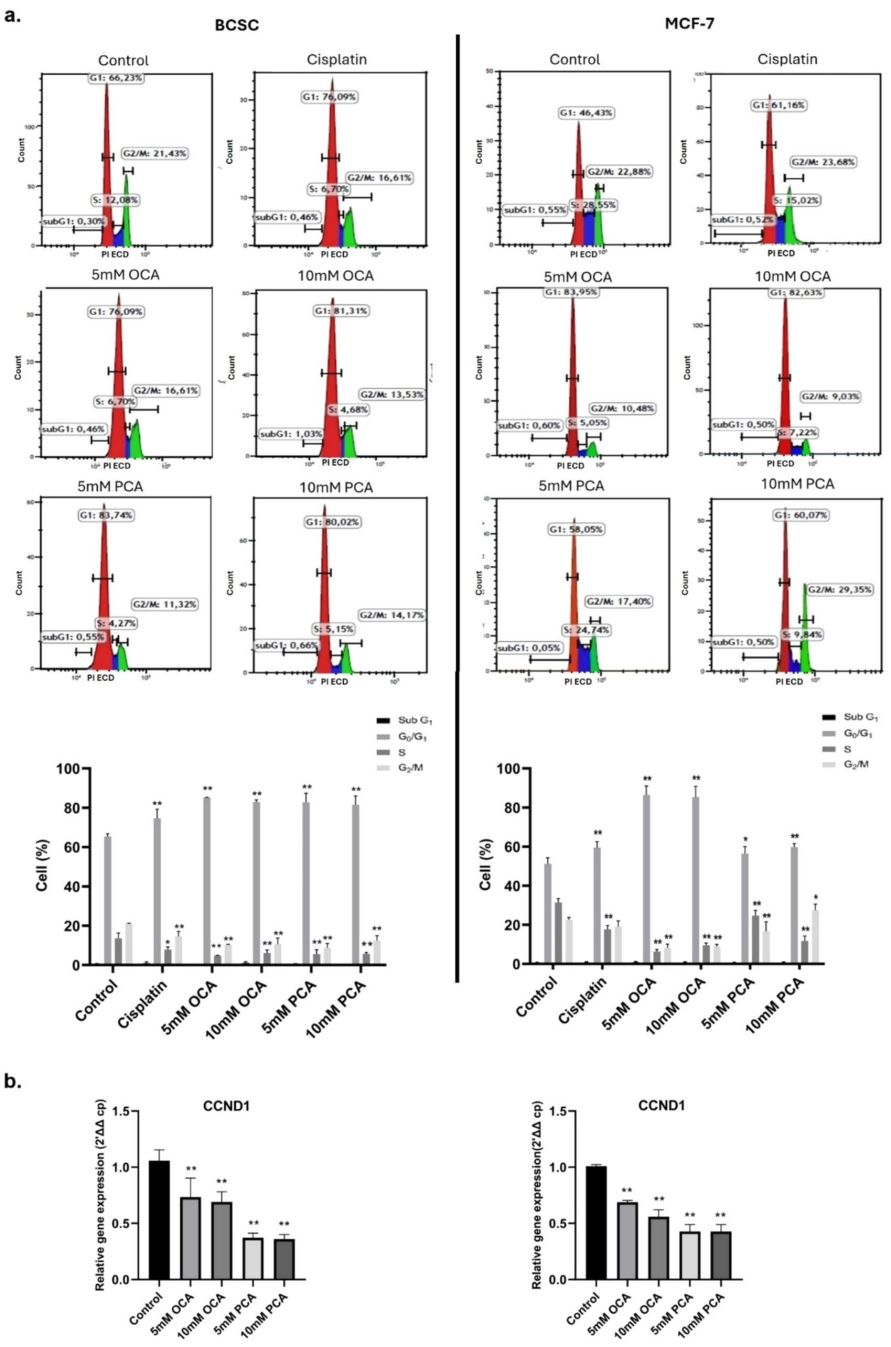
The effect of compounds on cell cycle profile (**a**). Cell cycle analysis of cells incubated with OCA (5–10 mM), PCA (5–10 mM), and Cisplatin for 24 h. The percentage of cells in the subG_1_, G_0_/G_1_, S, and G_2_/M phases was analysed using Kaluza 2.1 software (**b**). CCND1 gene expression levels in cells treated with the compounds for 24 h (n = 3). Statistical analysis was performed using GraphPad Prism version 8.3 software and *p < 0.05, **p < 0.01. All data are expressed as the mean ± SD of three independent experiments

Next, to examine the effect of OCA and PCA on the cell cycle profile of BCSCs and MCF-7 cells, 7ADD staining with flow cytometry was performed for 24 h. The results showed that both HCA treatments led to the accumulation of cells at the G_1_/S phase starting from 5 mM in each cell line (Fig. 4a). In addition, compared to control cells, decreased cyclin D1 levels were observed in OCA- and or PCA-exposed cell lines. These findings suggest that OCA and PCA have cytostatic activity in addition to their cytotoxic activity in BCSC and MCF-7 cells (Fig. 4b).

### HCAs trigger apoptosis in BCSCs and MCF-7 cells

**Fig. 5:**
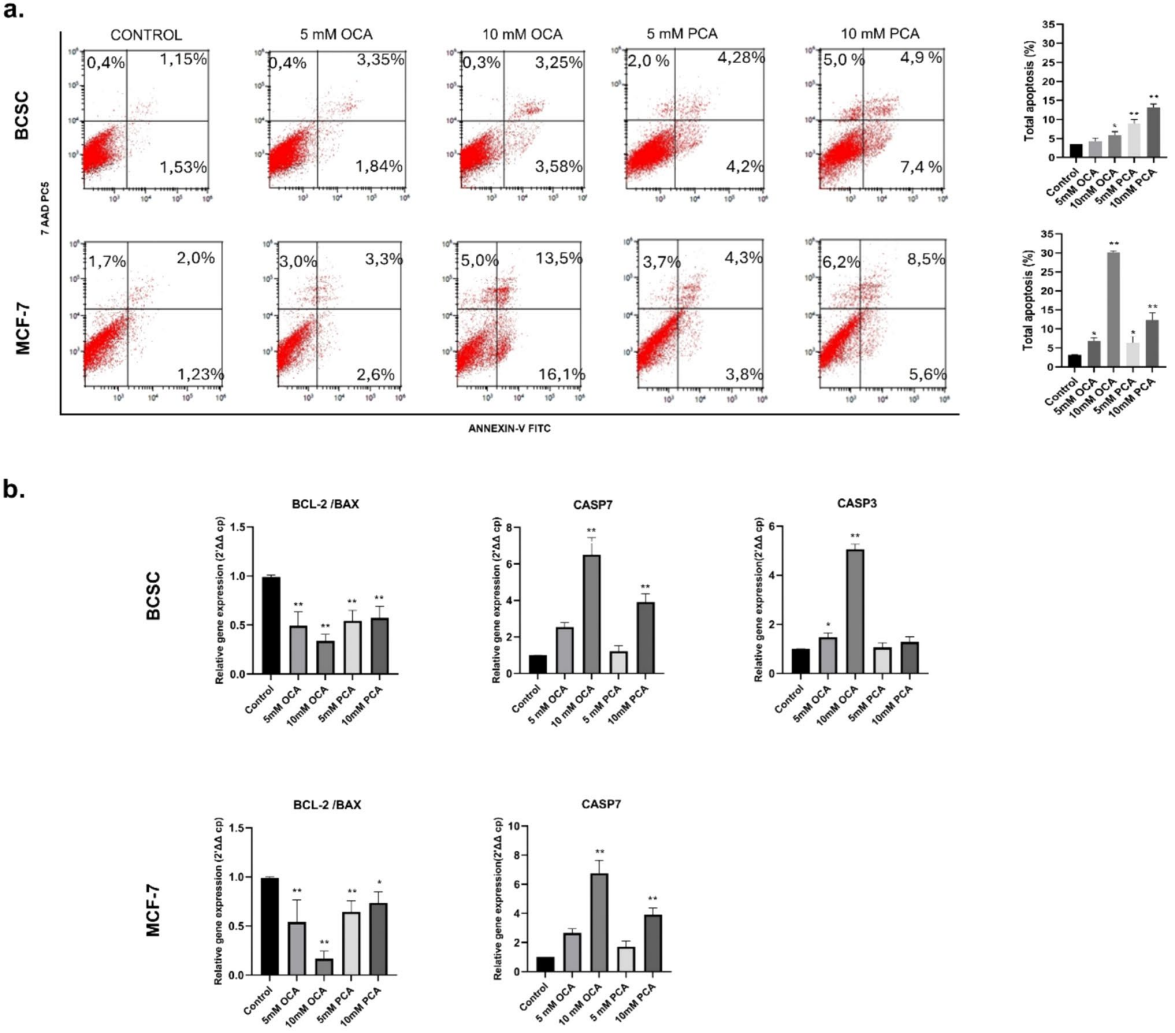
Evaluation of the effect on apoptosis of OCA and PCA in BCSCs and MCF-7 cells (**a**)**.** Flow cytometric analysis; the lower right region (Annexin-V positive,7AAD negative) indicates early apoptosis; the upper right region (Annexin-V positive,7AAD positive) represents late apoptosis and necrosis; the upper left region (Annexin-V negative,7AAD positive) denotes necrosis; and the lower left region (Annexin-V negative,7AAD negative) corresponds to viable cells. The total apoptosis rate was calculated as the sum of the early and late apoptosis rates (**b**)**.** RT-PCR analysis of apoptosis after cells were treated with the compounds for 24 h (n = 3). Statistical analysis was performed using GraphPad Prism version 8.3 software and *p < 0.05, **p < 0.01. All data are expressed as the mean ± SD of at least three independent experiments.

To investigate the ability of each HCA to induce apoptosis, cells were treated with 5 and 10 mM OCA and PCA for 24 h. Following Annexin-V- 7ADD staining, flow cytometry analyses were performed to determine the proportions of viable, early apoptotic, late apoptotic, and necrotic cells. The percentage of apoptotic cells increased significantly in PCA- and OCA-treated cells compared to that in the untreated controls. At a 5 mM concentration, total apoptosis rates were close, measuring 5.4 ± 1% for OCA and 8.3 ± 2% for PCA in both cell types.10 mM led to increased apoptosis rates of 6.83% for OCA and 12.3% for PCA in BCSC cells. Similar apoptotic profiles were observed after treatment of MCF-7 cells with 10 mM PCA. Interestingly, a dramatic increase in cell death was observed in MCF-7 cells treated with 10 mM OCA (Fig. 5a). Next, the alteration in gene expressions involved in apoptosis was examined after treatments for 24 h. As shown in Fig. 5b, the Bcl-2/Bax ratio decreased, and caspase 7 expression increased in cells treated with OCA or PCA at concentrations of 5 mM. However, a significant effect was observed in 10 mM OCA- and PCA-treated cells. Additionally, the elevation of the expression of the caspase-3 gene was detected after each treatment in BCSC but not in MCF-7 cells due to the lack of caspase-3 [28]. Interestingly, we did not detect significant caspase 3 expression after treatment with PCA, which might indicate that PCA-generated apoptosis did not involve caspase-3, but caspase-7. In summary, both agents promoted apoptosis, with 10 mM having the greatest effect on the expression of the apoptotic genes.

### OCA and PCA inhibited ERK1/2 activation but induced phosphorylation of JNK and p38 MAPK

**Fig. 6:**
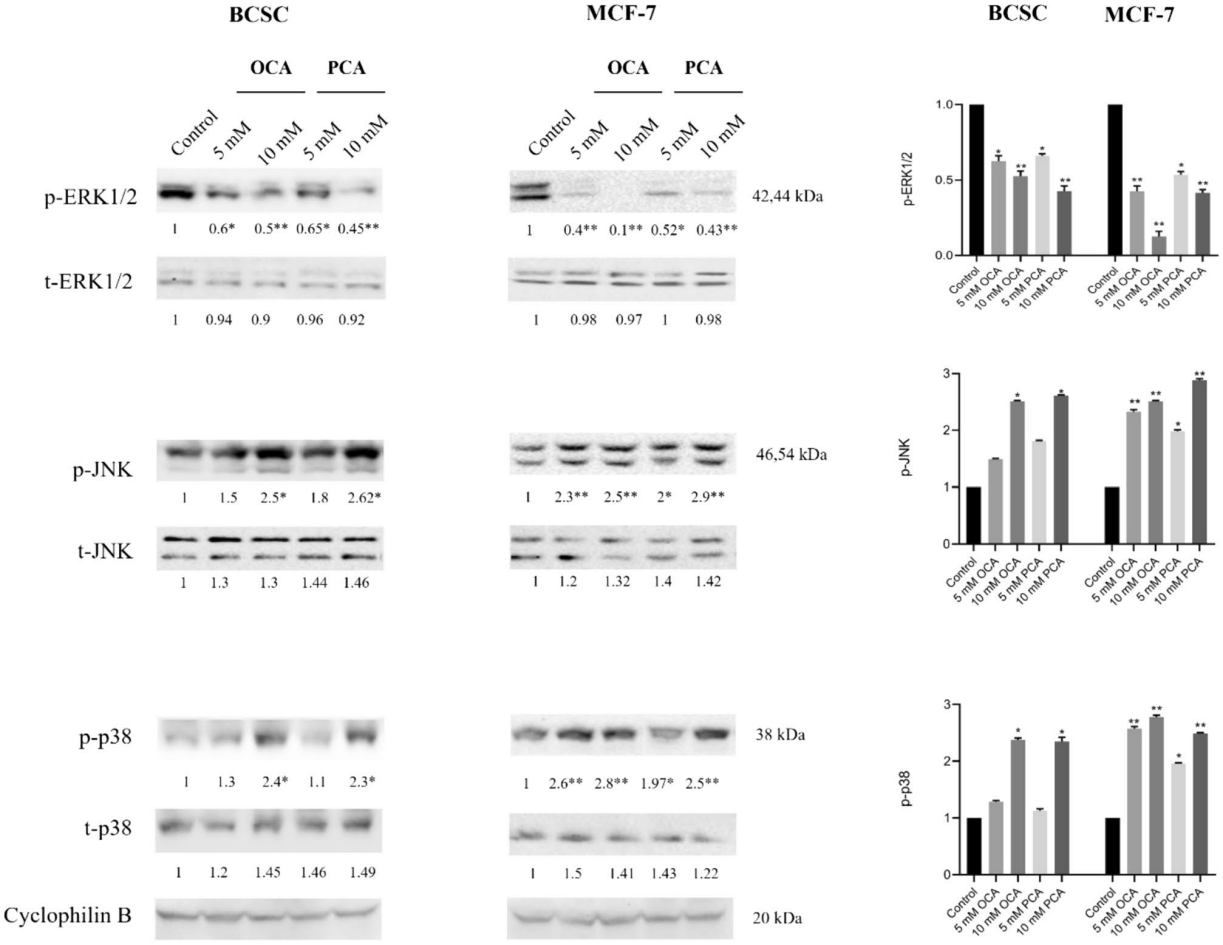
Expression profiles of proteins involved in MAP kinase pathway Cells were treated with 5- and 10 mM OCA and PCA for 24 h (n = 4). Western blot analysis was repeated at least three times, and statistical analysis was performed using Two-Way Anova *p < 0.05, and **p < 0.01

To evaluate the effect of OCA and PCA on MAPK signalling pathways, in which ERK1/2 activation was associated with cell proliferation signals but the JNK and p38 were frequently associated with apoptotic induction, western blot analysis was performed after both cells were treated with HCAs for 24 h. As shown in Fig. 6**,** both OCA and PCA significantly downregulated ERK1/2 phosphorylation in a concentration-dependent manner. This result demonstrated that OCA and PCA inhibited proliferation signals in BCSC and MCF-7 cells. In contrast, both compounds induced concentration-dependent increases in p-JNK and p-p38 levels in both cell types. Significant stress-activated protein phosphorylation altered in MCF-7 cells at 5 mM, but 10 mM in BCSCs might indicate an elevated sensitivity of MCF-7 cells to apoptosis.

## Discussion

Cancer stem cells, a subpopulation of tumor cells, have garnered attention in cancer treatment because they have been implicated in delaying treatment responses and driving metastasis and recurrence following therapy [29]. Several studies have been reported on the anti-cancer effect of PCA, the most abundant HCA derivative in nature, but what is known about its isomer, OCA, is limited [14–17, 20–23]. In this study, the anti-cancer effects of HCA isomers on BCSCs and MCF-7 were investigated in the concepts of growth/proliferation, cell cycle, and apoptosis.

It is well known that CD44 is a cell surface glycoprotein that serves as a specific receptor for hyaluronan and plays a crucial role in breast cancer cell adhesion, migration, and invasion [5]. CD44 is also involved in cell proliferation and tumor angiogenesis [6]. CD44 is linked to stem cell characteristics, whereas CD24 expression is associated with cancer cell differentiation [30]. Since the identification of BCSCs in 2003, the CD44^+^/CD24^−^ phenotype has been widely used as a reliable marker for their isolation [31]. In addition, high ALDH activity has been proposed as an important marker for identification [32]. ALDH, a family of cytosolic enzymes, oxidizes intracellular aldehydes and converts retinol to retinoic acid during stem cell differentiation [8]. Ginestier et al. demonstrated that ALDH1 + breast cancer cells can form tumors in the subcutaneous fat pads of NOD/SCID mice [32, 33]. In vivo and in vitro studies have identified CD49f and CD61 as BCSC markers [34]. CD133, another stem cell marker, supports tumor development, metastasis, and resistance to chemotherapy and radiotherapy [35]. In our study, flow cytometry analyses revealed that the cells isolated from the tumor tissue exhibited a CD44^+^/CD24^−^ phenotype, CD61 positivity, high ALDH enzyme activity, and 49% CD133 positivity. BCSCs demonstrated the ability to form mammospheres over two generations. According to Zhang et al., there are four main methods for identifying and isolating cancer stem cells: surface marker or biomarker combination separation, side population cell isolation with Hoechst 33342 staining, the aldefluor method, and mammosphere formation [36].

The IC_50_ values of OCA and PCA were determined to be approximately 5 mM for both cell types, which is consistent with previous data revealing the inhibition value of OCA in MCF-7 cells [23]. Conventional microscopic analysis showed that OCA application especially altered the morphology of epithelial cells to be rounded, where BCSCs were considerably shortened, and a considerable reduction in cell confluency was observed at 10 mM OCA.

Previously, it was shown that PCA methyl ester significantly reduced cell migration by nearly 40% in human umbilical vein endothelial cells (HUVEC) [37]. Similarly, OCA and PCA inhibited the wound closure of BCSC in a concentration-dependent manner. However, wound closure was achieved faster in HCA-treated BCSCs than in MCF-7 cells. This finding indicates that BSCS cells demonstrate greater resilience than MCF7 cells following exposure to HCAs, potentially maintaining their migratory capacity. OCA treatment led to an increase in E-cadherin mRNA levels in both cell types. Interestingly, PCA increased E-cadherin levels in BCSCs while reducing its expression in MCF-7 cells. Amiradabi et al. reported that epithelial-derived MCF-7 cells typically migrate in clusters owing to increased E-cadherin expression [38, 39]. One study showed that migration increased in EC96 cells derived from adenocarcinoma with epithelial morphology because of E-cadherin re-expression [40]. In the last decade, experiments have shown that cancer cells, instead of performing full EMT, perform partial EMT (pEMT) by gaining mesenchymal properties, particularly by preserving epithelial markers such as E-cadherin [41]. The inhibitory effect of PCA on colony formation was previously demonstrated in a study on colorectal adenocarcinoma cells [42]. In our experiments, OCA and PCA have significantly prevented colony formation of BCSCs at concentrations lower than 5 mM concentrations, suggesting that HCAs were as successful as chemotherapeutic cisplatin in inhibiting the formation of novel colonies.

The cell cycle profile of the cells was evaluated after HCA treatments. OCA has been shown to reduce the expression of cyclin D1 and CDK2 and induce CDKN2A cell cycle proteins in MCF-7 cells [23]. Our analysis showed that both HCA caused the accumulation of BCSCs and MCF-7 cells at the G1/S phase and downregulation of cyclin D1 expressions. These findings support the initial results and indicate the cytostatic effect of these compounds on BCSCs and MCF-7 cells.

AnnexinV-7AAD assay measurements in HCA-exposed cells demonstrated that 10 mM OCA stimulated apoptosis in BCSCs and MCF-7 cells. These assays were confirmed by the Bcl-2/Bax ratio and increased caspase expression. In these experiments, OCA produced a much stronger apoptotic response than PCA in both cell types. It increased the expression of caspase 7 by approximately twice the fold in both cell lines. Since caspase-3 expression was deficient in MCF-7 cells [28], we examined caspase-3 expressions only in BCSCs, where the expression level of caspase-3 was increased in OCA but not in PCA-treated cells. These data indicate that PCA-stimulated apoptosis of BCSC was caspase-3 independent. Both compounds induced apoptosis in both cell types, with 10 mM OCA demonstrating the highest apoptotic effect. Interestingly, despite some data indicating the absence of caspase-3 activation in MCF-7 cells, some researchers reported that 10 mM PCA increased the apoptosis rate and caspase 3 protein levels in MCF-7 cells after 24 h [43]. Moreover, Sen et al. showed a significant increase in the apoptotic proteins Bax and caspase 3 and a decrease in the anti-apoptotic protein Bcl-2 in MCF-7 cells after treatment with OCA [23].

The mitogen-activated protein kinases (MAPKs) play a pivotal role in cells, and the abnormality of these signals leads to the progression of various cancers, which makes them key therapeutic targets. The common downstream effector of classical MAPK, ERK1/2, is activated by mitogens and growth factors to promote cell proliferation, differentiation, and migration [44]. Our protein analysis showed that the ERK1/2-mediated MAPK pathway was inhibited after HCA treatment, confirming the suppressive effect of OCA and PCA on proliferation. PCA pretreatment inhibits LPS-induced phosphorylation of ERK1/2 and JNK in mouse macrophage RAW264.7 cells [17]. PCA has also been demonstrated to suppress EGFR gene expression, which is an essential effector of the RAS/RAF/MEK/ERK pathway, in HCT-15 colorectal adenocarcinoma cells [45, 46]. Our results showed that JNK and p38 were stimulated by OCA and PCA in BCSC and MCF-7 cells. The activation of these pathways is often associated with cytostatic and/or apoptotic mechanisms [47]. A significant increase in the phosphorylation of p38 and JNK proteins was observed when 10 mM OCA or PCA was applied to BCSCs; however, this induction was detected at lower concentrations in MCF-7 cells, which showed an elevated sensitivity of MCF-7 to HCA treatments. These results further highlighted that OCA and PCA affect MAPK signalling in a concentration- and cell-type-dependent manner, suggesting their potential as therapeutic agents to target the MAPK pathway. Furthermore, considering that JNK and p38 pathway activations are frequently associated with apoptosis, the activation of these MAPK modules by OCA and PCA may mediate the induction of apoptosis in our cells.

In conclusion, OCA and PCA were found to reduce the activity of primary BCSCs. Both HCAs inhibited growth and proliferation, arrested the cell cycle, and induced apoptosis, but OCA was more effective than PCA for these cells. These results suggest the need to further examine the mechanisms of action of PCA and OCA in BCSCs to support efforts to overcome treatment resistance in breast cancer.

## Data Availability

No datasets were generated or analysed during the current study.

## Abbreviations

BCSCs: Breast cancer stem cells
HCA: Hydroxycinnamic acid
PCA: Para-coumaric acid
OCA: Ortho-coumaric acid
ALDH: Aldehyde dehydrogenase
ERK1/2: Extracellular regulated kinase1/2
JNK: Janus kinase
p38 MAPK: P38 Mitogen-activated kinase
